# Efficient Structural Relaxation of Polycrystalline Graphene Models

**DOI:** 10.3390/nano11051242

**Published:** 2021-05-08

**Authors:** Federico D’Ambrosio, Joris Barkema, Gerard T. Barkema

**Affiliations:** 1 Department of Information and Computing Sciences, Utrecht University, Princetonplein 5, 3584 CC Utrecht, The Netherlands; G.T.Barkema@uu.nl; 2 Informatics Institute, University of Amsterdam, Science Park 904, 1098 XH Amsterdam, The Netherlands; jorisbarkema@gmail.com

**Keywords:** polycrystalline graphene, monte carlo simulation, graphene models

## Abstract

Large samples of experimentally produced graphene are polycrystalline. For the study of this material, it helps to have realistic computer samples that are also polycrystalline. A common approach to produce such samples in computer simulations is based on the method of Wooten, Winer, and Weaire, originally introduced for the simulation of amorphous silicon. We introduce an early rejection variation of their method, applied to graphene, which exploits the local nature of the structural changes to achieve a significant speed-up in the relaxation of the material, without compromising the dynamics. We test it on a 3200 atoms sample, obtaining a speed-up between one and two orders of magnitude. We also introduce a further variation called early decision specifically for relaxing large samples even faster, and we test it on two samples of 10,024 and 20,000 atoms, obtaining a further speed-up of an order of magnitude. Furthermore, we provide a graphical manipulation tool to remove unwanted artifacts in a sample, such as bond crossings.

## 1. Introduction

Graphene is a crystal of carbon atoms that form a three-coordinated honeycomb lattice. It is a material with a large set of exotic properties, both mechanical and electronic, and it has the particularity of being a two-dimensional crystal embedded in a three-dimensional space [1,2,3,4,5,6,7,8]. Large samples experimentally produced are usually polycrystalline, containing intrinsic [9,10,11], as well as extrinsic [12] lattice defects. These defects warrant a thorough study as they both have a significant detrimental effect on the properties expected from pristine graphene [13,14], and they can also cause new effects that are otherwise absent [15,16,17,18].

In particular, structural defects are both prominent and common in graphene [19], as they can easily host lattice defects due to the flexibility of the carbon atoms in hybridization. Such defects can be frozen in the sample during the annealing process and have been experimentally observed [20,21,22]. Their controlled production in graphene has been explored [23].

Since unsaturated carbon bonds are energetically very costly [19], polycrystalline graphene samples can be studied with the use of continuous random network (CRN) models [24], introduced by Zachariasen almost 90 years ago to represent the lack of symmetry and periodicity in glasses [25]. The rules of this type of model are quite simple: The only requirement is that each atom is always perfectly coordinated, i.e., their bonding needs are fully satisfied. Wooten, Winer, and Weaire (WWW) introduced an explicit algorithm to simulate the evolution of samples of amorphous Si and Ge, the so-called WWW algorithm that became the standard for this kind of model [26,27]. In the WWW approach, a configuration consists of a list of the coordinates of all *N* atoms, coupled with an explicit list of the bonds between them.

We opted for the empirical potential for polycrystalline graphene recently proposed by Jain et al. [24]:

$$E=\frac{3}{16}\frac{\alpha }{d^{2}}\sum_{i,j}{\left(r_{ij}^{2}-d^{2}\right)}^{2}+\frac{3}{8}\beta d^{2}\sum_{j,i,k}{\left(\theta_{j,i,k}-\frac{2\pi }{3}\right)}^{2}+\gamma \sum_{i,jkl}r_{i,jkl}^{2}$$

with $r_{ij}$ as the distance vector between the atoms *i* and *j*, $\theta_{j,i,k}$ as the angle centered on the atom *i* between the atoms *j* and *k*, $r_{i,jkl}$ as the distance between the atom *i* and the plane described by its neighbors j,k,l, and d=1.420Å as the ideal bond length of graphene. The other parameters, extracted from DFT calculations [24] are $\alpha =26.060e\;V/{Å}^{2}$, $\beta =5.511e\;V/{Å}^{2}$ and $\gamma =0.517e\;V/{Å}^{2}$. The interaction with the substrate on which the sample lays is simulated by a harmonic confining energy term in our potential

(1) $$E_{c}=K\;\sum_{i=1}z_{i}^{2}$$

where $z_{i}$ is the z-coordinate of the atom with index *i* and *K*, a prefactor that is determined empirically in order to constrain the maximum buckling height to the range of 4–8 Å, experimentally observed with scanning tunneling microscopy (TEM) [11].

The process starts with a completely random 2D sample with all atoms perfectly coordinated, in the case of graphene three-fold connected, generated with the Voronoi diagram algorithm described in [24]. In order to generate an initial configuration with *N* atoms, we place N/2 random dots in a 2D square box, which is then surrounded by 8 copies of itself to implement periodic boundary conditions. We then compute the *N* vertices of the Voronoi diagram [28] of these random dots, which will be replaced by atoms, and connect them along the edges of the diagram to form the bonds by them. This highly energetic configuration is then carefully relaxed with molecular dynamics.

The structure of the sample evolves through a series of bond transpositions involving four connected atoms with two bonds that are broken to create two new bonds. After each bond transposition, the system is relaxed; the move is accepted according to the Metropolis acceptance probability [29,30]:

(2) $$P\left(X^{\prime }|X\right)=\min\left\{1,\;\exp\left[\frac{E\left(X\right)-E\left(X^{\prime }\right)}{k_{b}\;T}\right]\right\}$$

where *X* and $X^{\prime }$ are the configurations of the system respectively before and after the bond transposition, both the coordinates and the list of bonds. $k_{b}$ is the Boltzmann constant, *T* is the temperature, and E(Y) the energy of the configuration *Y* after complete relaxation. Relaxing the sample, even with an optimized molecular dynamics algorithm such as the FIRE algorithm [31], has a significant computational cost, which is wasted if the bond transposition is ultimately rejected. As the energy of the sample is gradually lowered through bond transpositions, the accepted ratio becomes smaller, often well below one per cent, and almost all computational time is wasted on proposed bond transpositions that are eventually rejected.

Barkema and Mousseau [32] developed a method for amorphous silicon that allows the early rejection of bond transpositions before completing the relaxation of the sample. It generates a stochastic energy threshold beforehand, given by

(3) $$E_{t}=E_{b}-k_{b}\;T\;\ln\left(s\right)$$

where *s* is a random number between zero and one. In the first ten relaxation steps, the sample is relaxed only locally up to the third neighbor shell. The energy is assumed to be harmonic around the minimum; therefore, the final energy can be approximated as proportional to the square of the force

(4) $$E\left(X^{\prime }\right)\approx E-c_{f}{\left|F\right|}^{2}$$

where $c_{f}$ is an empirically determined constant, and *F* the force vector. Once we are close enough to the minimum, we can immediately reject the bond transposition if, at any moment during the relaxation, $E-c_{f}{\left|F\right|}^{2}>E_{t}$. The efficiency of this method is dependent on the quality of the assumption Equation (4); for amorphous silicon, the type of model for which it has been developed, this approximation is generally valid after just a few relaxation steps.

**Figure 1:**
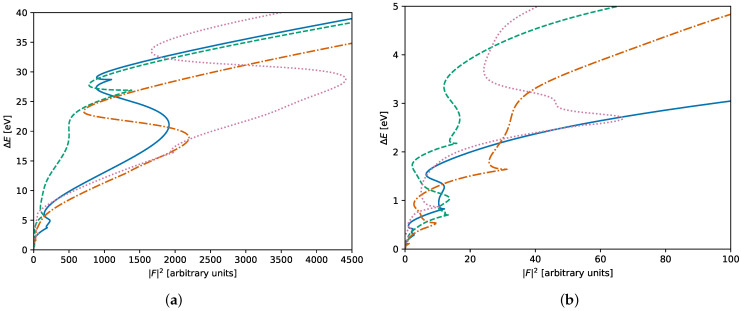
Some typical relaxation trajectories of a 3200 atoms sample, farther (**a**) and closer (**b**) to the origin. Even after thousands of iterations, the approximation of Equation (4) cannot be applied to this system as it fluctuates rapidly in the phase space. ΔE is the energy difference with the relaxed (final) energy, ${\left|F\right|}^{2}$ the magnitude of the forces.

In theory, this approach could also be applied to polycrystalline graphene. Unfortunately, the harmonic approximation of Equation (4) is only valid very close to the minimum; as we show in Figure 1, the trajectory of the system in the phase-space fluctuates rapidly and erratically during the relaxation, instead of following the expected linear relation between the excess energy and squared force magnitude after a certain number of relaxation steps. Without this approximation, a very costly full relaxation is necessary after each attempted bond transposition. A different approach is needed.

In this work, we propose a new method where only the atoms up to a shortest-path distance *l* from the atoms involved in the bond transpositions are initially allowed to relax. The energy of the sample after this local relaxation is then used to predict the final energy and immediately reject hopeless bond transpositions, without requiring a full relaxation. We test this approach on a 3200 atoms sample, comparing the performance for different values of *l*. The quality of the results is also compared to those obtained only through global relaxation. We further propose a variation of this method for relaxing large samples, and we test it by generating and relaxing a 20,000 atoms random sample.

## 2. Materials and Methods

The initial configuration of the sample is a disordered, perfectly three-fold coordinated, and two-dimensional random network. It is generated following the procedure described in [24].

The coordinates of the sample are relaxed to an energy minimum with molecular dynamics, following the FIRE technique [31]. After setting a temperature lower than the melting point of graphene, several bond transpositions are performed until it reaches reasonably low energy and a realistic configuration. Once this flat sample is sufficiently relaxed, every atom is placed at a random non-zero distance out of the two-dimensional plane and allowed to relax to a buckled three-dimensional configuration.

**Figure 2:**
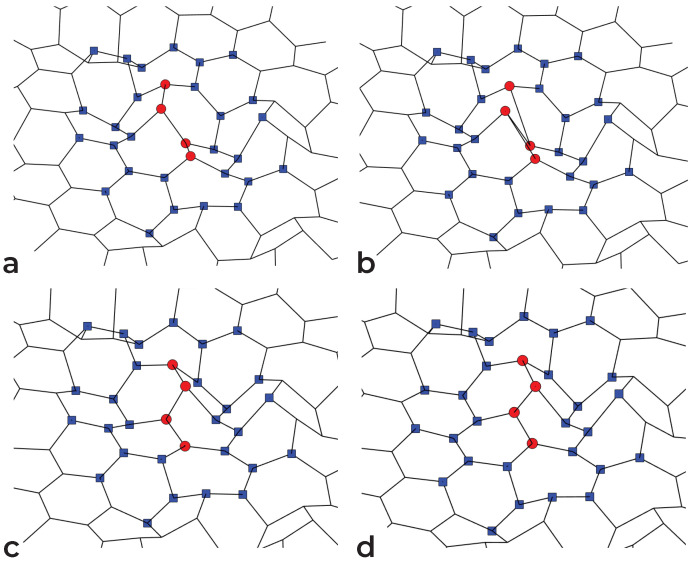
Successful bond transposition on a sample of graphene: (**a**) initial configuration; (**b**) bond transposition—atoms involved are marked with red dots; (**c**) local relaxation—atoms involved are marked with blue squares; (**d**) final configuration after global relaxation.

**Figure 3:**
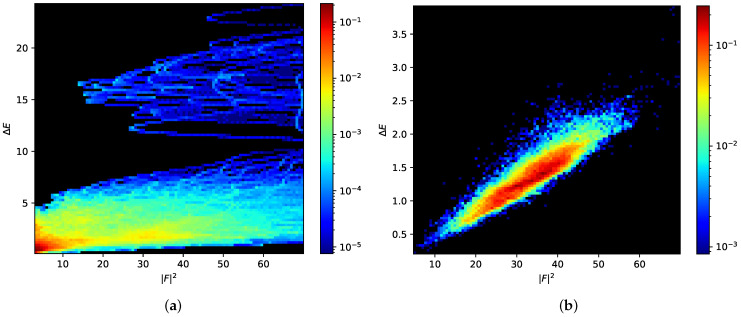
Two-dimensional histograms of the energy difference with the relaxed (final) energy ΔE and the force strength ${\left|F\right|}^{2}$ over many relaxations of a 3200 atoms sample, from the values assumed during global relaxation, (**a**), and at the end of local relaxation, (**b**). Frequency is show in logarithmic scale. We can see that the values assumed at the end of local relaxation follow the harmonic approximation of Equation (4).

Our approach to the structural relaxation of graphene can be followed in Figure 2. Four consecutive atoms are randomly selected (Figure 2a), and the bonds between the first two and the last two are transposed (Figure 2b). The energy threshold is computed from Equation (3), and we perform a local relaxation around the four atoms involved in the bond transposition; instead of limiting it to a certain number of relaxation steps, the atoms up to a shortest-path distance *l* from the transposed bonds are allowed to relax completely (Figure 2c). The list of atoms involved in the local relaxation is computed after each attempted bond transposition by iteratively exploring the network, starting from the four atoms involved in the bond transposition, and checking against duplicates. After local relaxation, attempted bond transpositions for which $E_{l}\left(X^{\prime }\right)-c_{f}{\left|F\right|}^{2}>E_{t}$, with $E_{l}\left(X^{\prime }\right)$ the energy of the sample after local relaxation up to distance *l*, are immediately rejected. In contrast with the method from [32], the criterion is applied only once, instead of at each point of the relaxation (with possibly some upper bound on the force strength). As we note in Figure 3, the force strength after local relaxation (Figure 3b) is a good estimator for the final energy, especially in comparison to the force strength during the global relaxation (Figure 3a), which is used by the method in [32].

### 2.1. Early Rejection

In the early rejection approach, the whole sample is otherwise allowed to relax (Figure 2d) and, if $E\left(X^{\prime }\right)<E_{t}$, the bond transposition is finally accepted. As less than one per cent of proposed moves are accepted in a relaxed sample, we expect the speed-up to be significant: Most are rejected after relaxing a limited number of degrees of freedom.

The value of $c_{f}$ is fine-tuned from empirical data collected from the simulation itself, targeting a higher bound on the rate of false negatives (i.e., bond transposition that are rejected erroneously), which in this work was fixed at 2% of the total number of attempts that should have been accepted. No transposition is accepted without complete relaxation, regardless of the result of the local minimization; therefore, no false positive (i.e., a bond transpositions is accepted erroneously) can be introduced by this technique.

### 2.2. Early Decision

While the computational time required for each local relaxation is constant with regard to the size of the sample, the same cannot be said for the global relaxation after each tentatively accepted bond transposition. For large samples, due to the amount of computational time that this requires, the structural relaxation still grinds almost to a halt. This is particularly problematic for the initial structural relaxation of a large random sample, which requires a large number of bond transpositions to reach a realistic, more relaxed state.

We propose, as an alternative for such cases, the early decision approach: The decision on whether to reject or accept a bond transposition after the local relaxation is treated as final, without having to perform a global relaxation to accept it. The parameter $c_{f}$ is still fine-tuned from empirical data but in this case, we opt for the value that best fits it. After a successful bond transposition, the system will not reach the energy it would have reached with global relaxation and the forces on atoms outside those involved in the last local relaxation will not go to zero. To correct for this issue, the energy threshold for accepting a bond transposition, see Equation (3), will be computed replacing the current energy of the system ($E_{b}$) with an estimation of the energy that our current configuration would reach after a global relaxation according to Equation (4). It can also be useful to set an upper value for the magnitude of the forces that, when reached, will trigger a global relaxation that will stop when the magnitude of the forces is comparable to those that are leftover after a single bond transposition, to reduce the time spent on these occasional global relaxations.

It must be noted that since we are replacing the energy of the relaxed system after a bond transposition in Equation (2) with an estimate, the early decision method does not guarantee detailed balance, as opposed to the early rejection method. Nevertheless, this method is extremely powerful when performance is more critical than accuracy; for instance, for the structural relaxation of a very large randomly generated sample when it is still far away from equilibrium. In these cases, detailed balance is not as critical and a large number of bond transpositions are required to reach a state closer to the equilibrium.

### 2.3. Manipulation Tool

The initial random configuration can incorporate artifacts such as two bonds crossing each other. While in most cases these defects will gradually disappear as the sample relaxes to a more ordered configuration, some artifacts might be particularly resilient and can persist even when the sample is otherwise sufficiently relaxed. Such defects have to be removed manually. We have developed a graphical tool called Graphene Editor (available online: https://github.com/jorisBarkema/Graphene-Editor, accessed on 13 March 2021) to facilitate this work. This tool allows the user to upload and download a sample, explore it visually, add and remove bonds, move one or more atoms, replace a single atom with three connected atoms and vice-versa and check the consistency of the sample over the number of bonds for each atom and bond crossings.

## 3. Results

### 3.1. Early Rejection

A random sample with N=3200 atoms was generated following the procedure described in the previous section. WWW bond transpositions are performed until the sample is relaxed to reasonably low energy, approximately 625 eV (less than 0.2 eV/atom). The values of $c_{f}$, seen in Table 1, for different values *l* of the local relaxation radius are chosen empirically, with the constraint of keeping the ratio of false negatives (successful bond transpositions that are nevertheless rejected) over successful bond transpositions under 2%, while still rejecting a large part of unsuccessful moves. The quantity $c_{f}$ is expressed in units of seconds squared over the atomic mass unit ($s^{2}\;u^{-1}$). The average number of atoms involved in the local relaxation for different values of *l* is also shown in Table 1.

**Figure 4:**
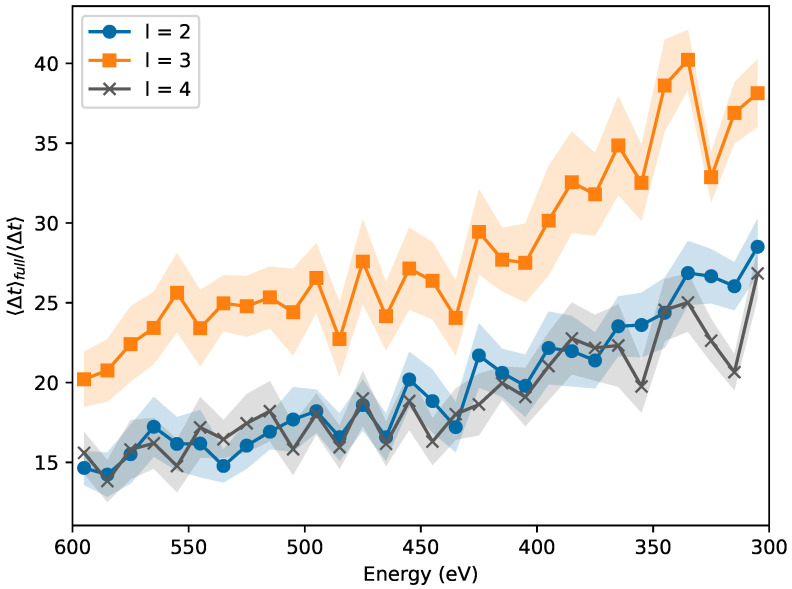
Average speed-up per accepted bond transposition, as ratio between CPU time required with full relaxation and early rejection, for different values of *l*: blue dots (2), orange squares (3) and grey crosses (4). The shaded area shows one standard deviation from the average. The speed improvement grows as the sample becomes more crystalline and its energy lowers, while best results are obtained for l=3, with an improvement of a factor between 20 and 40.

Starting from the same initial sample, we perform bond transpositions both using the usual WWW algorithm with full minimization and the early rejection method proposed here, with different values of *l*. The temperature is set to T=3000K for both samples. After each successful bond transposition, we record the energy, the elapsed time in central processing unit (CPU) clocks, and the number of attempts since the last successful move. The simulation is stopped once the system reaches a final energy of $E_{f}=200\;e\;V$, equivalent to 0.0625 eV/atom. At least ten relaxation cycles are performed with the early decision method (with different values of *l*) and with complete relaxation after each bond transposition. As we note in Figure 4, the average CPU time per accepted bond transposition is improved by at least an order of magnitude. The speed-up grows as the sample grows larger crystalline domains and more random attempts are necessary per accepted bond transposition. The best results are obtained for l=3, which leads to an efficiency improvement of a factor between 20 and 40.

**Figure 5:**
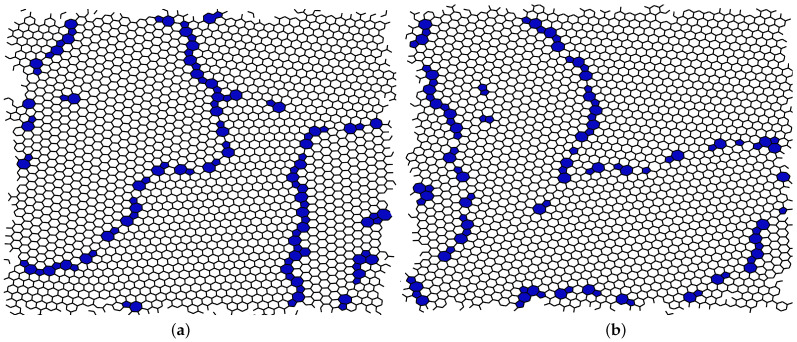
Final configurations of the sample at E≈200 eV obtained (**a**) through only global relaxations; (**b**) through our local relaxation method with l=3. Highlighted in blue are the defective (i.e., non-hexagonal) rings. The two samples are qualitatively indistinguishable: Same level of separation between crystalline domains of similar sizes.

The early rejection method does not alter the amount of relaxation obtained at the end of the process. As we note in Figure 5, both the level of separation between crystalline domains, i.e., the degree to which the defects are present on the borders between them, and the size of the domains are consistent. The ring statistics of the two final configurations, computed with the Ring Statistics Algorithm (available online: https://github.com/vitroid/CountRings, accessed on 13 March 2020) [33] and reported in Table 2, are also consistent. In this final configuration, the ratio of false negatives is lower than 0.5%.

### 3.2. Early Decision

As we noted in the previous section, while the early rejection technique is quite powerful for most samples, it is insufficient for very large samples; our attempt to relax a very large sample (N = 20,000) could not reach our initial energy target of 1 eV/atom after more than a month, due to the computational time required by each global relaxation that takes place at least once per accepted bond transposition. In the early decision method, the decision on whether to accept a bond transposition or not takes place directly after performing a local relaxation, based on the estimated relaxed energy of the sample.

We relaxed with both approaches a randomly generated sample of 20,000 atoms. We opted again for l=3 for the local relaxation, and $c_{f}$ is set after fitting the data from ĩ00 global relaxations. The force magnitude thresholds are set in such a way that a global relaxation should be triggered each 50–100 successful bond transpositions and stopped when the force magnitude reaches a value comparable with what is usually left after just one local relaxation. The temperature is set to T=3000 K.

**Figure 6:**
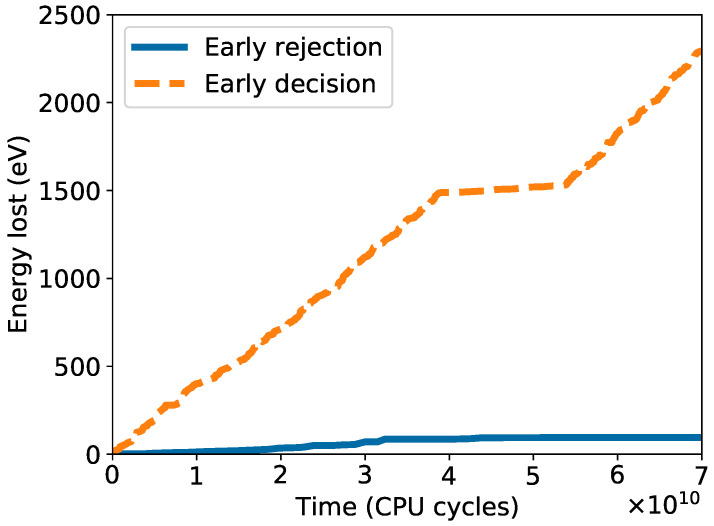
Structural relaxation of a large, randomly generated sample (N = 20,000), starting from an energy of 1.15 eV/atom with early rejection (blue solid line) and with early decision (orange dotted line) methods. The early decision method performs significantly faster, reaching a speed-up of a further order of magnitude. We also notice a plateau around $0.5\times 10^{11}$ CPU cycles in the early decision line where, due to the forces accumulated from previous bond transpositions, our algorithm was incorrectly rejecting bond transpositions, slowing the evolution of the sample significantly.

We initially performed the relaxation on a sample with energy of 1.15 eV/atom. As we can see in Figure 6, the early decision approach leads to a significant speed-up that we estimate to be around one further order of magnitude. The speed-up factor per bond transposition is stable during the relaxation at approximately 22. Both methods accept, on average, a bond transposition every seven attempts, but the early decision method is, as expected, less stable: There can be phases where it is not able to correctly estimate the correct decision to take. In these extreme cases, bond transpositions are erroneously rejected and the evolution of the sample slows down. This is especially the case when the magnitude of forces accumulated from previous bond transpositions become significantly large. We can see such a case in the plateau of the orange dotted line in Figure 6, and it underscores the importance of setting a correct threshold for the magnitude of forces accumulated before triggering a global relaxation.

**Figure 7:**
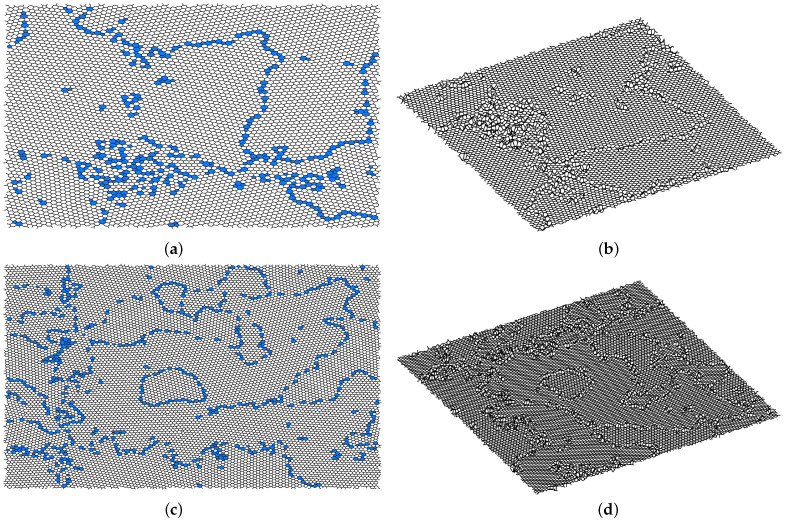
Final configurations of the 10, 024 atoms sample in two (**a**) and three (**b**) dimensions and the 20,000 atoms sample in two (**c**) and three (**d**) dimensions, obtained through early decision local relaxation with l=3. Defects (i.e., non-hexagonal rings) are highlighted in blue in the two-dimensional plots and clearly visible due to the buckling in the three-dimensional plots. The two samples are qualitatively very similar to those of Figure 5, with large domains surrounded by defects.

Finally, we relaxed the 20,000 atoms sample and another sample of 10,024 atoms down to 1488.05 eV (0.074 eV/atom) and 695.51 eV (0.066 eV/atom), respectively. The temperature is initially set at 3000 K and then gradually reduced, in order to reach lower energies. The resulting samples, as we note in Figure 7, present large crystalline domains with defects accumulating on their boundaries, similar to Figure 5. As we note in Table 3, both samples have reached similar ring statistics, with less than 10% of defected rings and only a handful (less than 0.2%) defected by more than one atom (i.e., octagons). The ring statistics are computed with the Ring Statistics Algorithm [33].

All the samples presented in this paper are available online: (https://github.com/federicodambrosio/graphene-samples, accessed on 20 March 2021).

## 4. Discussion

In summary, we introduced two techniques that, through local relaxation, can estimate the success of bond transpositions reducing or eliminating the need for relaxing the entire sample, which is extremely time-consuming. Both techniques significantly reduce the computational time required per accepted bond transposition: The early rejection method by immediately rejecting, without a global relaxation, hopeless attempts; the early decision method avoids global relaxations entirely, relying on the estimate of the energy of the relaxed sample.

The early rejection technique should be preferred for average-sized samples, especially if already well-relaxed since it gives an already significant speed-up while it guarantees that the dynamics are not compromised. Furthermore, its accuracy also improves as the energy of the sample is reduced. The early decision technique leads to an even larger speed-up but does allow for attempts to be erroneously rejected and should therefore be used when performance is a priority above accuracy, for instance when the sample is still very far from equilibrium and detailed balance is less critical. Since thousands of bond transpositions are required to reduce the energy of a few hundreds of electron volt, the cumulative speed-up obtained through either of these techniques can easily reach multiple orders of magnitude. These techniques open up the possibility of generating larger random samples with ordinary computers in an affordable amount of time.

Finally, our manipulation tool Graphene Editor makes those small manipulations that are often necessary as simple and quick as they can be.

## Figures and Tables

**Table 1:** Empirically determined values of the harmonic coefficient $c_{f}$ and average number of atoms involved in local relaxation $\langle N_{loc}\rangle$ for different local relaxation distances *l*, in a sample of size N=3200 atoms.

*l*	$c_{f}$ [s${}^{2}$ u${}^{-1}$]	$\langle N_{\mathrm{loc}}\rangle$
1	$3.21\times 10^{-3}$	13
2	$4.63\times 10^{-3}$	28
3	$5.33\times 10^{-3}$	53
4	$9.08\times 10^{-3}$	90

**Table 2:** Ring statistics for the two final configurations of the 3200 atoms sample, relaxed with full relaxation (left) and Early Rejection (right). We note that they both have reached similar statistics, with around 93% of the rings being hexagons, 3–4% heptagons and pentagons, while octagons are too rare at this energy to compare between the two.

Atoms	Full Relaxation		Early Rejection	
**Size**	**#**	**%**	**#**	**%**
5	56	3.50	60	3.75
6	1489	93.06	1480	92.50
7	54	3.38	60	3.75
8	1	<0.01	0	0.00

**Table 3:** Ring statistics for the two large samples of 10,024 and 20,000 atoms. We note that they both have reached similar statistics, with over 90% of the rings being hexagons, 4–5% heptagons and pentagons and less than 0.2% octagons.

Atoms	10,024		20,000	
**Size**	**#**	**%**	**#**	**%**
5	231	4.6	487	4.87
6	4554	90.86	9043	90.43
7	223	4.45	453	4.53
8	4	0.08	17	0.17

## Data Availability

Data available on request to authors.
